# The Dangers of Opium in Mania a Potu

**Published:** 1868-09

**Authors:** J. O. Whitney

**Affiliations:** Pawtucket, R. I.


ART. II.—*The Dangers of Opium in Mania a Potu.* BY J. O. WHITNEY, M. D., *Pawtucket, R. I.*

The following case of death, produced by one teaspoonful of laudanum, shows the care and judgment required when using this potent, but most useful drug:

J. C., a rugged man, about fifty years of age, had been stimulating freely during the last half of June, 1868, and, as usual with him, such course entirely destroyed his appetite for food. Wakefulness ensued, for which laudanum was procured and used in teaspoonful doses by the family, without medical advice. This condition of things getting no better, but probably worse, as on the evening of July 3d, "the room was full of people" to him. The above quantity was given in whisky, between six and seven o'clock, when he retired to bed. In three hours thereafter his breathing attracted attention, and he was lifted into a chair; not awaking by midnight, I was called to see him. He had the aspect of a dying person; pulse nearly or quite gone at the wrist, ashy pale lips, a profuse cold perspiration throughout, lividity of finger-nails; the breathing did not indicate immediate death from narcotism, still it was by no means normal; pupils contracted. The family was at once informed that he would die, when rough measures were proposed; flagellation, walking him about, and the like modes of arousing the patient. It seemed to me these would at once put out the feeble spark of life existing. In view of all the points in the case, I recommended that nothing should be done, for the chance to save his life by any means known, had long passed. He expired a little after one o'clock on the morning of the 4th, an hour or thereabouts after I first saw him. Death was caused by the drug paralyzing the heart; the exhausted state of the vital powers consequent on his protracted stimulation and fasting, being the reason so small a quantity ended in disaster. Every fact connected with the administration of the laudanum was carefully inquired into, and nothing could be elicited but that one teaspoonful only had been given that day; the vial containing it was inspected, and presented no evidence of its having been above ordinary strength.

Relating this case to a practitioner of much experience, he gave full credence to the statement of the family, saying that he had long since abandoned the use of opium in delirium tremens as a dangerous remedy, having had three patients that died where he had used it; and he said "the drug paralyzes the heart in such cases." My patient certainly did not die from asphyxia, as I have usually seen when opium had been taken in a fatal dose.

Two years since a man of great powers of life took half a drachm of morphine for the purpose of destroying his life. He was seen by me in about five hours; the heart was beating with strength, but the respirations were reduced to three, five, and up to eight or ten per minute, and very irregular, seeming at times that the last breath had been taken. At these times the heart would almost cease to act, but its action never became feeble, however slow and faltering, the beat always denoted strength, and the face was livid, (instead of ashy paleness.) In this case the drug paralyzed respiration, and the indication was clear, to wit: to maintain respiration until the poison of the morphine passed off. This we apparently effected by electricity, for when the breathing became the worst the fluid passed from the nucha to the epigastrium would arouse action, and after thus maintaining it for four or five hours, consciousness returned. I think the man fairly owes his life to electricity, notwithstanding his powerful heart, for few men could withstand thirty grains of morphine unaided. Of late large doses of digitalis have been given in delirium tremens; this drug is now said to be a heart tonic; may it not be that the debility of the heart, which is inseparably incident to constant stimulation, requires these large quantities of digitalis?

I have seen a stout child, eight months old, destroyed with three or four teaspoonfuls of "Mrs. Winslow's Soothing Syrup," given at the ordinary intervals, at the commencement of cholera infantum. In this disease I use anodyne, with extreme caution, if at all; it is destructive to a child in collapse in such ailment. That one drachm of ordinary laudanum should kill a rugged man seems incredible, and had the death taken place by paralyzing the respiratory apparatus, I should have placed no confidence in the statement. The mode of death, coupled with the assertion of the physician above referred to, are noteworthy points.

